# Educational Effectiveness, Target, and Content for Prudent Antibiotic Use

**DOI:** 10.1155/2015/214021

**Published:** 2015-04-05

**Authors:** Chang-Ro Lee, Jung Hun Lee, Lin-Woo Kang, Byeong Chul Jeong, Sang Hee Lee

**Affiliations:** ^1^National Leading Research Laboratory of Drug Resistance Proteomics, Department of Biological Sciences, Myongji University, 116 Myongjiro, Yongin, Gyeonggido 449-728, Republic of Korea; ^2^Department of Biological Sciences, Konkuk University, 1 Hwayangdong, Gwangjingu, Seoul 143-701, Republic of Korea

## Abstract

Widespread antimicrobial use and concomitant resistance have led to a significant threat to public health. Because inappropriate use and overuse of antibiotics based on insufficient knowledge are one of the major drivers of antibiotic resistance, education about prudent antibiotic use aimed at both the prescribers and the public is important. This review investigates recent studies on the effect of interventions for promoting prudent antibiotics prescribing. Up to now, most educational efforts have been targeted to medical professionals, and many studies showed that these educational efforts are significantly effective in reducing antibiotic prescribing. Recently, the development of educational programs to reduce antibiotic use is expanding into other groups, such as the adult public and children. The investigation of the contents of educational programs for prescribers and the public demonstrates that it is important to develop effective educational programs suitable for each group. In particular, it seems now to be crucial to develop appropriate curricula for teaching medical and nonmedical (pharmacy, dentistry, nursing, veterinary medicine, and midwifery) undergraduate students about general medicine, microbial virulence, mechanism of antibiotic resistance, and judicious antibiotic prescribing.

## 1. Introduction

Immunologist Frank Macfarlane Burnet in 1953 predicted the “virtual elimination of infectious disease as a significant factor in social life” [1]. The opinion of microbiologist Ernest Jawetz, as expressed in his manuscript published in 1956, was also similar: “On the whole, the position of antimicrobial agents in medical therapy is highly satisfactory. The majority of bacterial infections can be cured simply, effectively, and cheaply. The mortality and morbidity from bacterial diseases has fallen so low that they are no longer among the important unsolved problems of medicine. These accomplishments are widely known and appreciated” [2]. However, since the last decade of the 20th century, the emergence and spread of antibiotic resistance in pathogenic bacteria, including staphylococci, pneumococci, and Gram-negative species, pose an emerging threat to public health [3]. This threat is increased by the decline of novel antibiotics introduced in the market [4, 5]. Therefore, the most effective strategy for combating antibiotic resistance is the decrease of antibiotic use and prudent antibiotic prescribing.

However, misuse or overuse of antimicrobial agents is still prevalent and acts as a significant contributor to the emergence of antibiotic resistance [6]. Antimicrobial drugs are unique drugs that directly affect the growth of microbial pathogens, instead of affecting the patient. Antibiotic prescribing is a complex process which requires considering the characteristics of pathogens as well as the characteristics of a patient and a drug. Nevertheless, most clinicians can prescribe antibiotics without any regulation or certification, while only specialists in oncology can prescribe anticancer drugs [6, 7]. Prescribers of antibiotics such as physicians and pharmacists encounter dual, somewhat contradictory responsibilities. On the one hand, they want to provide optimal therapy for their patients and this responsibility tends to promote an overuse of antibiotics. On the other hand, they have a responsibility to future patients and to public health in sustaining the efficiency of antibiotics and minimizing antibiotic resistance; however, this responsibility can be easily ignored [6]. A lack of knowledge of prescribers in epidemiology and antibiotics can trigger maximal broad-spectrum treatment. Therefore, educational efforts targeted at antibiotic prescribers are certainly required.

Antibiotic resistance is no longer just a hospital problem [8]. The community managed by local healthcare providers, as distinguished from specialist hospitals or regional centers, also plays an important role in the emergence and spread of antibiotic resistance [6, 9]. The World Health Organization (WHO) has emphasized the importance of the public within the community as well as professionals in the control of antibiotic resistance [8]. Several studies have shown that the adult public's overexpectation about the activity of antibiotic against pathogens increased the number of antibiotic prescription [10, 11]. Many adult people still have serious misunderstandings about antibiotics (e.g., antibiotics are useful for colds) [6]. Children's elementary knowledge of antibiotic use is also poor [12], despite the highest antibiotic prescription rate of children group [8].

Therefore, the multifaceted approach that combines education of children, campaigns for the adult public, and antibiotic stewardship programs for medical professionals should be conducted. In this review, we will examine the importance of educating prescribers and the public (adults and children) and discuss some relevant aspects, including the content of teaching programs, training the trainer, and evaluation of program effectiveness.

## 2. Methods

**Figure 1:**
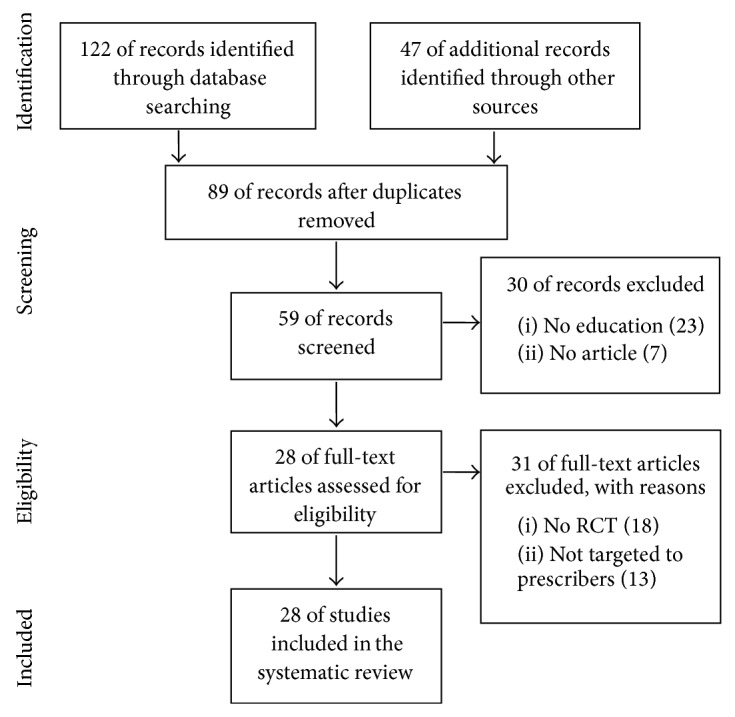
Literature selection process (PRISMA flow diagram).

To assess the effectiveness of clinician education, we used the Preferred Reporting Items for Systematic Review and Meta-Analysis (PRISMA) [13] in our review (Figure 1). We conducted a systematic literature search in the following databases: Medline via PubMed and the Cochrane Library. Additionally, we also searched on the following websites: National Institute for Health and Care Excellence (http://www.nice.org.uk), Canadian Agency for Drugs and Technologies in Health (http://www.cadth.ca), Current Controlled Trials (http://www.controlled-trials.com), and BioMed Central (http://www.biomedcentral.com). We used keywords as search terms. We combined terms for selected indications (education, antibiotic, and randomized controlled trial). The literature search included all studies published in English between 1983 and 2014. We identified 28 randomized controlled trial (RCT) studies containing clinician education. In addition, recent articles pertaining to educational programs for teaching prudent antibiotic use were also examined.

## 3. Effectiveness of Education about Prudent Antibiotic Use

To examine the effectiveness of educational strategies, we searched a variety of studies assessing the effectiveness of education programs that currently work. Because most educational efforts were focused on clinician education, we first searched RCT studies assessing the effectiveness of clinician education programs alone or combined with other strategies, using the Preferred Reporting Items for Systematic Review and Meta-Analysis (PRISMA). We identified 28 RCT studies containing clinician education. Most studies showed that reduction in antibiotic prescribing was achieved through interventions focused on clinician education programs, such as interactive seminars [14], mailing campaigns [15, 16], small-group education focusing on evidence-based medicine and communication skills [17–19], educational outreach visit [20–24], guidelines and leaflets [25–30], and a combination of these educational strategies [31, 32]. On average, antibiotic prescription of the intervention group was reduced by 34.1% (from 9% to 52%) compared with the control group. The number of inappropriate antibiotic prescriptions was also more reduced by 41% on average than the control group. These results indicate that clinician education can significantly improve antibiotic prescribing.

It is hard to compare effectiveness between educational strategies and to conclude definitely which educational intervention shows better outcome, because of the differences between the participants' characteristics. Nevertheless, when effectiveness between educational interventions was compared, reduction rates of antibiotic prescribing through educational programs are as follows: interactive seminars, 25%; mailing campaigns, 9%; small-group education, 52%; educational outreach visit, 30%; guidelines and leaflets, 42%; combination, 23%. Therefore, the intervention containing small-group education seems to be most often effective. Despite these results, the effectiveness of clinician education without combining with other strategies remains controversial, because several studies have shown no significant effect on antibiotic prescription after education [33, 34].

To minimize misuse or overuse of antibiotics in hospital, besides clinician education, there are a number of intervention programs, such as patient education, delayed prescriptions, audit and feedback, clinician reminder and decision support system, and financial and regulatory incentives or disincentives. Various studies to evaluate the effect of multifaceted interventions combined with other strategies such as audit and feedback intervention have also been published. The effectiveness rates of combined education intervention were tested in combination with other strategies, including patient education [35, 36], delayed prescriptions [37], audit and feedback [38–42], and clinician reminder and decision support system [17]. Multiple interventions decrease the rate of antibiotic prescription to 72% compared with the control group [41] and seem to be more frequently effective than interventions using education strategy alone [34]. Reduction rates of antibiotic prescribing of education interventions in combination with other strategies are as follows: patient education, 14%; delayed prescriptions, 45%; audit and feedback, 72%; clinician reminder and decision support system, 57%. Clinician education in combination with audit and feedback intervention seems to be the most effective way. Therefore, these results show that clinician education combined with other strategies can more effectively decrease the rate of antibiotic prescription (compare the maximum reduction rates of clinician education alone (52%) and in combination with other strategies (72%)).

Because human behaviors are affected by complex factors, such as attitudes and beliefs, a close collaboration with behavioral and social sciences is required when developing an educational intervention program [6]. Furthermore, because human behaviors can change over time, it is also important that the educational messages are repeated routinely. A recent report about long-term effects of an educational seminar on antibiotic prescribing by clinicians showed that a standardized and interactive educational seminar results in a long-term reduction in antibiotic prescribing and could justify a large-scale implementation of this intervention [14]. In summary, for effective implementation of ASPs, education intervention is required to be combined with active strategies, such as prospective audit and feedback.

In contrast to extensive studies about the effectiveness of clinician education, there were only several studies about the effect of educational programs for undergraduate medical students, the public, or children. Unfortunately, there is no RCT study assessing the effectiveness of educational programs for these groups. Furthermore, as far as we know, there is no study measuring the effectiveness of an educational program on prudent antibiotic prescribing for medical students. There have been several formal evaluations of public campaigns aimed at improving antibiotic prescribing. Some successful campaigns directed to the general population have led to a substantial reduction in prescribing in Australia [43, 44], the United States [45–47], and Europe [48–51]. However, in another report, there was no improvement in antibiotic use after the national antibiotic campaign [52]. Systematic reviews showed that multifaceted interventions involving both physicians (active education through seminars and visits) and the public/patients (through written material and mass media) seem moderately more effective than single interventions, in decreasing unnecessary antibiotic use [53, 54]. These reports suggest that public campaign through written material and mass media could be more effective if physicians facilitate the transmission of information about antibiotic use to the public including patients [55].

Several studies aimed to evaluate the effectiveness of school educational programs in improving student's knowledge of judicious antibiotic use. In the Czech Republic, France, and England, the study to evaluate the efficacy of the e-Big teaching pack showed that the junior e-Bug teaching pack demonstrated a significant improvement in student's knowledge in all sections, and this knowledge was retained 6 weeks later [12]. However, in the senior e-Bug pack, student knowledge improvement varied between regions, although consistent improvement in all sections was observed in England and Czech Republic [12]. In Portugal, the efficacy of the school intervention to improve the knowledge of middle school students of prudent antibiotic use was evaluated [56]. Although a lack of knowledge among students regarding antibiotic use was found in the pretest, significant improvements in knowledge were observed after implementation of the teaching activity. To enhance student's understanding of conceptions and processes underlying antibiotics' production and activity, such as the notion of action mechanisms of antibiotics, a hands-on interventional program containing a set of wet and dry laboratory activities was developed for high school students [57]. The data from surveys of 42 high school students support that this program promotes more sophisticated conceptualizations of bacteria, antibiotics, and antibiotic resistance [57]. However, to draw a clear conclusion for the effectiveness of educational programs for the public and children, a RCT study including the large number of students treated is required.

In conclusion, many RCT studies demonstrated that clinician education to reduce antibiotic prescribing is fairly effective, regardless of the type of educational programs. However, there is no RCT study assessing the effectiveness of educational programs for the public or children. This may be caused by the fact that up to now most of the effort has been put into the development and assessment of educational programs for physicians in hospitals. Therefore, for the development of effective educational programs for other groups as well as physicians, we will summarize current educational programs and investigate the missing aspect of educational programs for prudent antibiotic use.

## 4. Summary and Improvement of Current Educational Strategies for Prudent Antibiotic Use

### 4.1. Target Groups

**Figure 2:**
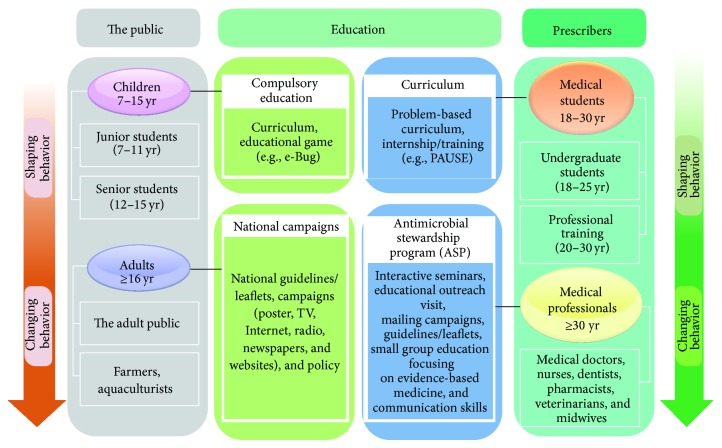
Education of prescribers and the public on prudent antibiotic use.

Figure 2 shows target groups of education about prudent antibiotic use. They can be divided into two groups, prescribers and the public. The prescribers of antibiotics include all healthcare professionals in contact with patients (e.g., medical doctors, undergraduate students, nurses, dentists, pharmacists, and midwives) and the veterinarians prescribing antibiotics for animals. The public can be divided into two groups, children and adults.

A lack of knowledge about antibiotics may significantly affect the quality of prescribing [6]. Nevertheless, unlike many other drugs, which are prescribed by a specialty, antibiotics are prescribed universally by almost any clinician without any regulation or certification. Junior doctors often prescribe antibiotics under the supervision of their seniors. In some countries, midwives, clinical pharmacists, or nurses can also prescribe some antibiotics in selected clinical situations [58]. Therefore, all healthcare professionals in contact with patients, including junior doctors, nurses, pharmacists, and midwives, must receive continual education about antimicrobial resistance. In particular, as most of antibiotics are used in primary care, continuous training on prudent antibiotic prescribing in primary care is important.

A recent WHO report emphasizes the importance of undergraduate training courses in prudent prescribing of antibiotics [59]. In some countries, including the United Kingdom (UK), education about prudent antibiotic prescribing is included as a component of the undergraduate curriculum [60]. The undergraduate curriculum could include education about microbiology, infectious diseases, and clinical pharmacology, with emphasis on prudent antibiotic prescribing [61].

We must also consider the antibiotic prescriptions for animals and agriculture. Antibiotics have also been used in veterinary medicine since the first commercial antibiotic became available for the treatment of human disease [6]. Although some antibiotics are designed exclusively for veterinary use, most antibiotics being used in veterinary medicine belong to the same antimicrobial classes as those being used for human diseases [62]. Recent studies reported the presence of extended-spectrum *β*-lactamase- (ESBL-) producing and carbapenemase-positive* Enterobacteriaceae* strains in food animals [63, 64]. These antibiotics are critically important to human therapy [6]. The use of antibiotics in veterinary medicine needs to be reduced. Therefore, the veterinarians, farmers, and aquaculturists should also receive education about antimicrobial resistance.

Because perceived patients have an increasingly participative role in the decision to prescribe in general practice [65, 66], education of the adult public including patients is also important. Several studies showed that the adult public remains unaware of elementary knowledge of antibiotic use and frequently engages in misinformed behaviors [67, 68].

Because children have relatively more creative inquiry-based activities to promote active learning, all junior (7–11 years) and senior (12–15 years) groups who need to undergo compulsory education are the most appropriate target group for education about prudent antibiotic use. Although most countries have the curricula covering the topic of human health and hygiene, limited information about prudent antibiotic use and limitations of antibiotics were provided within compulsory education. However, many recent programs in Europe and the United States have been directed to the education of children [8, 12, 61].

### 4.2. Content of Education

#### 4.2.1. Education of Healthcare Professionals

Up to now, considerable effort has been put into education of physicians in hospitals. To optimize antimicrobial therapy and reduce antimicrobial resistance, many institutions conduct antimicrobial stewardship programs (ASPs) which are executed by multidisciplinary antimicrobial utilization teams comprising physicians, epidemiologists, pharmacists, microbiologists, and infectious disease specialists [7]. ASPs are a multifaceted approach categorized as educational, restrictive, and supportive (Table 1). Education of prescribers is an essential element of ASPs.  Table 2 presents the main elements for educating prescribers in ASPs, including general medicine, immunological and genetic host factors, microbial virulence, pharmacokinetic and pharmacodynamics properties of antibiotics, and basic knowledge of epidemiology. Although educational interventions are more popular among clinicians than restrictive interventions, without restrictive or supportive interventions, conducting passive education alone in a hospital has had a little effect on changing prescribing practices for antibiotics [69]. Because hospitals are complex institutions, the development of more effective educational sources, such as face-to-face and one-on-one educational sessions provided by physicians, is required.

In view of educational postgraduate course, training the prescribers of antibiotics in the community is also important. Internship/foundation training or close collaboration between local healthcare providers and academicians can give the opportunity for educating the prescribers in the community. For example, an open-access curriculum containing a series of postgraduate courses and workshops has been developed in the context of the European Union funded research project “Genomics to combat resistance against antibiotics in community-acquired lower respiratory tract infections in Europe (GRACE)” [70]. The website has a total of 153 presentations and 104 webcasts focused mainly on diagnostics and much less on therapy. In 2010, the European Centre for Disease Prevention and Control (ECDC) chose hospital prescribers as target for the* European Antibiotic Awareness Day* and provided the toolkit containing template materials and evidence-based educational key messages (http://ecdc.europa.eu/en/eaad/Pages/Home.aspx).

Some studies suggest that educational outreach visits using an academic detailer to meet face-to-face with the health professional in the community can be effective [23, 71]. The role of the academic detailer is to reinforce the educational material and emphasize alternatives to the prescription of a particular antibiotic [72]. However, because the long-term sustained efforts and expense are required to implement and maintain ASPs in the hospital, education for the prescribers in the community can sometimes be easily ignored. Therefore, if education about prudent antibiotic prescribing is started at the time of undergraduate, when knowledge, attitude, and behavior of healthcare professionals are being shaped, the burden for educating the prescribers in the community can be significantly decreased.

#### 4.2.2. Education of Undergraduate Students

Until now, little attention has been given to the education of medical students. Recently, some reports evaluating medical students' perceptions, attitudes, and knowledge about antimicrobial prescribing and resistance have been published. In the United States, 92% of 317 medical students agreed that strong knowledge of antimicrobials is important in their careers, and 90% said that they would like more education about appropriate prescribing of antibiotics [73]. Similarly, in the UK, the study has demonstrated that medical students desire more education focused on antimicrobial prescribing [74]. The significant differences between the types of educational resources which medical students use to learn about antimicrobial prescribing and resistance were also identified. Notably, students who referred to infectious disease specialists, pharmacists, or IDSA guidelines as the sources of educational information had statistically significant higher knowledge scores compared with students who did not use those resources, whereas students who referred to Wikipedia as the educational source had lower knowledge scores [73]. Therefore, the development of the formal curricula of medical schools about antimicrobial use and resistance is required.

Rather than formal lectures, interactive learning in the format of problem-based learning with case vignettes can be suitable for this topic. For example, the University of Nijmegen (Netherlands) offers a program-based module on antibiotic policy for third year students, treating the history of infectious diseases, hygiene and infection control, antibiotic guidelines, and principles of prophylaxis [61]. In addition, standardized educational electronic tools establishing easy access to accurate information could improve knowledge of antimicrobial prescribing in undergraduate students. In the United States, through collaboration with the Centers for Disease Control and Prevention (CDC) and the University of Minnesota, Michigan State University developed an open-access learning site for veterinary medical students aimed at teaching the prudent antibiotic prescribing in veterinary practice [75]. In the UK, the Prudent Antibiotic User (PAUSE, http://www.pause-online.org.uk), a website of shared standardized teaching materials for prudent antimicrobial prescribing, was developed for the undergraduate medical curriculum [61].

#### 4.2.3. Education of the Adult Public

A number of research reports investigated the level of knowledge, attitude, and behavior regarding antibiotic of the general public around the world [76–79]. In Italy, only 9.8% of the respondents knew the definition of antibiotic resistance and 21.2% knew when it was appropriate to use antibiotics [79]. In Sweden, 19.1% of the respondents agreed that antibiotics can be used to treat coughs and colds successfully; this belief was higher in those who had not previously received antibiotics. The respondents showed some confusion surrounding the terms “bacteria” and “viruses” and the meaning of these terms in relation to the decision to prescribe [76]. Similarly, although the UK has been ranked as the fourth lowest prescriber in Europe [80], reports showed that 30% of English adults wrongly believe that antibiotics cure common colds more quickly [68, 81]. These reports suggest that effective public education initiative should provide practical and appropriate aims and means to facilitate prudent antibiotic use by the public [79].

In last decade, to improve the public's knowledge of antibiotics, various national campaigns through several media (TV, radio, newspapers, posters, websites, etc.) have been directed to educate the public. For example, the ECDC has annually conducted the* European Antibiotic Awareness Day* since 2008 (http://ecdc.europa.eu/en/eaad/Pages/Home.aspx). In the United States, the CDC's* Get Smart: Know When Antibiotics Work* has been conducted (http://www.cdc.gov/getsmart/) as well. These public campaigns can contribute to the habit of more careful use of antibiotics in outpatients, at least in high-income countries [82]. Several systematic reviews showed that educational interventions focused on the appropriate use of antibiotics in the public can be successful especially when local context and barriers are adequately analyzed and addressed [53, 54]. In particular, multifaceted interventions involving both physicians (active education through seminars and visits) and the public (passive education through written material and mass media) seem moderately more effective than single interventions [51, 54]. However, reliable indicators about the efficacy of most of these programs targeting the general public are still missing [48].

#### 4.2.4. Education of Children

In many countries, antibiotic prescribing rates for children are the highest [8]. A report in 1999 showed that 55% of children aged 0–5 years in the UK received an average of 2.2 prescriptions each year for a *β*-lactam antibiotic [83]. In the UK, although antibiotic prescribing to children has decreased recently, there are still approximately 6 million antibiotic prescriptions for children each year [84]. The major causes of childhood illnesses are viral upper respiratory tract infections [84] and the spread rate of these illnesses can be reduced simply by implementing proper hand hygiene practices, regardless of antibiotics prescribing [85].

Programs directed to children education have been developed as follows: e-Bug in Europe, Do Bugs Need Drugs? in Canada, and the Microbes en question in French. The e-Bug, the European commission-funded antibiotic teaching resource [86], is a representative educational program for children. Since the launch in 2009, visits to the e-Bug site have increased annually and were recorded from 190 different countries [87]. The number of available languages has also increased from 8 to 25, with extension outside the EU into Turkey and Saudi Arabia [88].

The e-Bug resource aims not only to educate children about prudent antibiotic use, but also to educate them about hygiene and the spread of infection, thereby allowing them to protect themselves from many common childhood illness through appropriate hand and respiratory hygiene [81]. This teaching program has two interactive teacher-resource packs to assist in educating children aged 9–11 years (junior) and 12–15 years (senior), each comprising nine distinct lesson plans [12]. An introduction to microbes and hand and respiratory hygiene is taught through fun activities [8]. Because food-related infectious intestinal disease is extremely common, the importance of hygiene during food preparation is taught in junior schools, through making a chicken salad and observing how far they have spread harmful microbes. Within the senior school pack, the importance of safe sex is reinforced by demonstrating how easily a sexually transmitted infection can spread through unprotected sexual intercourse. Both packs also cover information on the body's natural defenses to fight infection, antibiotic use, and vaccinations.

To reinforce learning, many of the core messages in the senior school pack are the same as in the junior school pack, but the senior school pack also incorporates different activities and explores the issues in much greater depth. Furthermore, to allow for a range of abilities within a school, or in home-based work, extension activities are always included in each lesson plan [8]. To provide the artwork suitable for each school group, the junior school pack features cartoon microbes and children's artwork, whereas the microbes in the senior pack are more realistic and the activities are more factual and research-based, to link with the higher national curriculum requirements [8].

To distribute free e-Bug packs to all schools across Europe and beyond, all the pack materials can be downloaded directly at the e-Bug website (http://www.e-Bug.eu). This website also contains video clips of pack activities, student computer games, and presentations for teacher use. The junior game was developed as a platform game and the senior game was developed as a story-based detective game [89]. In the junior platform game, participants learn about harmful or useful microbes, hygiene, and antibiotics, through funny games, such as taking photographs of microbes, washing microbes away, making bread or yogurt, and using antibiotics as a bomb for killing both good and bad bacteria, but not viruses. The senior game consists of four missions, each comprising five stages using problem-based learning and learning outcomes. Four missions cover hand and food hygiene, antibiotic resistance and how it can be related to antibiotic overuse, and the importance of taking antibiotics as instructed [8].

Peer-education, defined as “the teaching or sharing of health information, values and behaviors by members of similar age or status,” has become an increasingly popular and trusted method for health promotion and prevention [90]. These programs have been mostly used for work with young people, based on the assumption that the young person's peer group has a strong influence on the way he or she behaves. Young people feel that their peers can be trusted and are a credible source of information and that they learn from and influence each other, as much in risky as in safe behaviors [91]. Peer-education initiatives are currently being taken forward as a joint research project between Public Health England and the Environmental Health Department [88]. To improve student knowledge of key health message, the project to produce a series of “train the trainer” materials aimed at informing school nurses and others about how to educate school children on hygiene, the spread of infection, and prudent antibiotic use, has also been piloted [88]. Parental beliefs, fears, and expectations have an influential voice in consulting the medicines used by children [92]. Therefore, a recent concern of e-Bug about educating parents through children is also important and interesting [93].

### 4.3. Training the Trainer

In hospitals, a multidisciplinary core group, including infectious-disease's specialists, microbiologists, pharmacists, and antibiotic stewardship team, must be involved in the development and implementation of a local educational program on prudent antibiotic prescribing [61]. A multidisciplinary core group in university hospitals must also be involved in the development of a curriculum for undergraduate medical students. However, a national training program for antibiotic stewardship trainers is the most effective strategy for training hospital educators. The project “ABS International: implementing antibiotic strategies for appropriate use of antibiotics in hospitals in member states of the EU” started in September 2006 as a training program for national antibiotic stewardship trainers in nine European countries (Austria, Belgium, the Czech Republic, Germany, Hungary, Italy, Poland, Slovenia, and Slovakia) [94]. This program provided participants with standard tools for implementing an antibiotic stewardship program in their hospitals (e.g., guidelines for treatment and surgical prophylaxis, organizational measures, and tools to analyze consumption data). The activity of the international study group is also important. The European Conference on Microbiology and Infectious Diseases (ESCMID) Study Group for Antibiotic Policies (ESGAP) has been organizing educational workshops on antibiotic stewardship for more than 10 years (http://www.escmid.org/esgap). ESGAP has published its effort in making an inventory of easily accessible available websites, which may be helpful to anyone who is willing either to implement or to improve hospital-based antimicrobial stewardship programs or to educate hospital professionals about better antimicrobial practices [61, 95]. ESGAP has also been biannually conducting postgraduate international education courses “Antimicrobial Stewardship: Measuring, Auditing, and Improving,” and over the past decade, this program has been completed with over 400 medical doctors, scientists, and clinical pharmacists trained.

School teachers and nurses delivering the educational sessions on prudent antibiotic use must also be trained. On a national level, a policy for educating school teachers and nurses should be required. For example, Public Health England is currently producing a series of “train the trainer” materials aimed at informing school nurses and others about how to educate school children about hygiene, the spread of infection, and prudent antibiotic use [88]. Participants of “train the trainer” workshop will be able to access training sources and other tools on the e-Bug website.

## 5. Discussion

Until now, most educational efforts have been targeted at medical professionals (mostly medical doctors) after their training and at the adult public [61]. In the past few years, educational efforts have been focused on the development of teaching programs to educate children. The recent WHO report highlighted that education of medical students on prudent antimicrobial prescribing is also an important part of antimicrobial resistance stewardship [59]. Once medical doctors become qualified, it is difficult to change their deeply established views and behaviors [96]. Notably, several survey studies about knowledge and attitudes of medical students showed that many students wanted further education about antibiotic prescribing [73, 74, 97, 98]. Therefore, it seems obvious that education about prudent antibiotic prescribing should be started at the undergraduate training track. However, although inclusion of education about prudent antibiotic use in the undergraduate curriculum has been achieved to various extents in some countries [61, 75, 99–101], education about prudent antibiotic prescribing in the medical school curricula requires commitment from medical schools on a national level to agree that antimicrobial stewardship is among the necessary skills to practice [61]. It is necessary that, besides the undergraduate curriculum in medicine, correspondent educational courses are included in nonmedical curricula of pharmacy, nursing, midwifery, dentistry, and veterinary medicine. They can also prescribe some antibiotics in selected clinical situations [58].

A 24-item electronic survey of fourth year medical students at the three medical schools (the University of Miami, the Johns Hopkins University, and the University of Washington) in the United States showed that the majority of students did not know the appropriate management of complicated urinary tract infections or antimicrobial-resistant infections [73]. Only half of the respondents properly recognized* Clostridium difficile* infection and the spectrum of activity of commonly used antibiotics. These results suggest that there are deficiencies in the medical school's curricula and addressing these deficiencies by standardization of medical school's curricula is required [73]. Development of an open-access learning site with accurate information could improve antimicrobial use. Although many medical students used Wikipedia as an educational source, students who used non-peer reviewed Internet-based sources such as Wikipedia had lower knowledge scores [73].

Basically, the main educational elements for prescribers described in Table 2 can be applied both for the undergraduate curriculum of medical doctors and teaching other healthcare professionals with some modifications. However, clinical decision-making is a complex course affected by various situations such as patient's demand. Therefore, in the undergraduate curriculum, problem-based learning or vignette-based clinical scenario teaching could be a more effective method than classical formal lectures. As mentioned above, in some countries, the undergraduate medical curricula with a problem-based module were developed [61, 75]. The skill to communicate with patients in situations of diagnostic uncertainty should also be included in the curriculum, to reduce the number of unnecessary prescribing [97]. For example, in the United States, Wake Forest School of Medicine, the CDC, and the Association of American Medical Colleges (AAMC) recently developed and piloted an antimicrobial stewardship curriculum for use in medical schools (http://www.wakehealth.edu/AS-Curriculum). This curriculum contains materials for the preclinical and clinical years of instruction. For the clinical years, the curriculum contains five small-group activities that highlight antibiotic stewardship principles through case-based scenarios and management of common infections where antibiotics are often misused [102].

## 6. Conclusions

To manage antibiotic resistance, continuous efforts to educate people about judicious antibiotic use are important. Up to now, most educational efforts have been targeted to medical professionals and to the adult public. However, because medical professionals and adults have already established their knowledge, attitudes, and behaviours about antibiotic use, it is difficult to change their deeply established views and behaviours. Thus, in the past few years, a variety of programs to educate children has been made. Many studies showed that these educational efforts targeted to medical professionals, the adult public, and children are fairly effective in improving serious misunderstandings about antibiotics and reducing its use. However, novel antibiotics introduced in the market are still rare and pathogenic bacteria are adapting quickly to existing antibiotics, particularly to antibiotics acting on the Gram-negative bacteria [6]. Therefore, educational efforts to reduce antibiotic use must be expanded. In this review, we discussed the importance of educating prescribers and the public, the contents of teaching programs, training the trainer, and evaluation of program effectiveness. Efforts on a national level to improve current educational programs are required and it is necessary to develop appropriate educational programs targeted specifically to each group. In addition, appropriate curricula to teach medical and nonmedical undergraduate students should be developed as soon as possible. Because the undergraduate training track is the time when knowledge, attitudes, and behaviours of medical professionals are being shaped, educating them about prudent antibiotic prescribing will be significantly effective in minimizing antibiotic resistance.

## Figures and Tables

**Table 1:** Antimicrobial stewardship strategies.

Strategies	Interventions
Educational programs	(i) Guidelines and leaflets (ii) Interactive seminars (iii) Educational outreach visit (iv) Mailing campaigns (v) Small group education focusing on evidence-based medicine and communication skills (vi) Patient education

Restrictive programs	(i) Audit and feedback (ii) Delayed prescriptions (iii) Clinical rounds discussing cases (iv) Compulsory antibiotic order form (v) Regulating contacts with the pharmaceutical industry (vi) Financial and regulatory disincentives

Supportive programs	(i) Multidisciplinary antimicrobial stewardship team (ii) Financial and regulatory incentives (iii) Computer-assisted management program (iv) Clinician reminder and decision support system (v) Therapeutic drug monitoring service

**Table 2:** Educational content on prudent antibiotic prescribing for prescribers.

Topic	Concept	Learning outcomes
Antibiotics	Modes of action of antibiotics	(i) Broad- or narrow-spectrum of antibiotics (ii) Basic principles of pharmacokinetics (PK) and pharmacodynamics (PD) properties of antibiotics (iii) Antimicrobial susceptibility test (iv) Basic principles of antibiotics cycling or mixing (antibiotic heterogeneity)
	Toxicity	(i) Collateral damage of antibiotic use (toxicity)
	Costs	(i) Lack of development of new antibiotics

Bacterial resistance	Mechanism of antimicrobial resistance	(i) Acquired resistance-mechanism of antibiotic resistance in pathogenic bacteria (ii) Intrinsic resistance-mechanism of antibiotic resistance in commensal bacteria (e.g., risk of *Clostridium difficile* infection or *Candida *spp. infection) (iii) Epidemiology of antibiotic resistance (iv) Importance of the appropriate use of antibiotics to minimize the emergence of resistance

Diagnosis of infection	Infection and inflammation	(i) Infection versus inflammation (ii) Proper use and interpretation of bacterial Gram stain, point-of-case tests (e.g., urine dipstick, streptococcal rapid antigen diagnostic test in tonsillitis), serology, and biomarkers of inflammation (e.g., C-reactive protein (CRP) and procalcitonin) (iii) Establishment of standardized diagnosis criteria for specific infections (e.g., community-acquired pneumonia, hospital-acquired pneumonia, cystitis, and pyelonephritis) (iv) Importance of taking microbiological samples for culture before starting antibiotic therapy
	Antimicrobial susceptibility testing (AST)	(i) Detection of antibiotic resistance (e.g., phenotypic method, PCR-based techniques, mass spectrometry, and microarrays)

Infection prevention and control	Clinical microbiology and indication for antimicrobials	(i) The nature and classification of pathogenic microorganism (ii) Clinical situations when not to prescribe an antibiotic (iii) Viral infections (iv) The importance of understanding the differences between colonization and infection (v) The principles and practices of the prevention and control of infection (vi) Transmission mechanism of pathogenic bacteria in both community and hospital setting (vii) Definition and indications of directed therapy versus prophylaxis (viii) Surgical antibiotic prophylaxis: indication, choice, duration, and timing (ix) The principles and benefit of vaccines
	Hygiene	(i) The importance of hand hygiene (ii) Transmission mechanism of pathogenic bacteria in hospital setting (iii) Prudent use of invasive devices, such as intravenous or urinary catheters, and incision and drainage of abscesses

Prescribing antibiotics	Empiric therapy	(i) Not initiating antibiotic treatment in the absence of bacterial infection (ii) Knowledge of when not to prescribe antibiotics and use alternatives (iii) Knowledge of when to use a delayed antibiotic prescription (iv) Choosing the dose and interval of administration based on PK/PD properties of antibiotics (v) Choosing the route of administration (intravenous versus oral) (vi) Estimating the duration of treatment (vii) An understanding of common side effects such as allergy
	Targeted therapy using diagnostics	(i) Reassessment of the antibiotic prescription (ii) Streamlining/deescalation based on microbiological results (iii) Decision to switch agent (narrow/broader spectrum, intravenous/oral) (iv) Therapeutic drug monitoring to ensure adequate drug levels (e.g., vancomycin) (v) Stop prescribing antibiotics when *Clostridium difficile* infection is present (vi) Reconcile and adjust antibiotics at all transitions and changes in patient's condition (vii) An awareness of trade and generic names of prescribed antibiotics to avoid possible harm to patients
	Antimicrobial stewardship program	(i) Prudent antibiotic prescribing according to national/local practice guidelines (ii) Avoiding the unnecessary use of broad-spectrum antibiotics (iii) Documentation of antibiotic prescription in clinical chart or in patient's clinical records: indication, route, dose, duration, and review/stop date of antibiotics

Communication skills	Discussion technique with patients	(i) Knowledge of when not to prescribe antibiotics and how to negotiate this with patients
